# Cortical BOLD responses to moderate- and high-speed motion in the human visual cortex

**DOI:** 10.1038/s41598-018-26507-0

**Published:** 2018-05-29

**Authors:** Kyriaki Mikellidou, Francesca Frijia, Domenico Montanaro, Vincenzo Greco, David C. Burr, Maria Concetta Morrone

**Affiliations:** 1 Department of Translational Research on New Technologies in Medicine and Surgery, University of Pisa, Pisa, Italy; 2 Unit of Neuroradiology, Fondazione CNR/Regione Toscana G. Monasterio, Pisa, Italy; 3 Istituto Nazionale di Ottica, CNR, Florence, Italy; 4 Department of Neuroscience, Psychology, Pharmacology and Child Health, University of Florence, Florence, Italy; 5 Neuroscience Institute, CNR, Pisa, Italy; 6 Stella Maris Scientific Institute, Pisa, Italy

## Abstract

We investigated the BOLD response of visual cortical and sub-cortical regions to fast drifting motion presented over wide fields, including the far periphery. Stimuli were sinusoidal gratings of 50% contrast moving at moderate and very high speeds (38 and 570 °/s), projected to a large field of view (~60°). Both stimuli generated strong and balanced responses in the lateral geniculate nucleus and the superior colliculus. In visual cortical areas, responses were evaluated at three different eccentricities: central 0–15°; peripheral 20–30°; and extreme peripheral 30–60°. “Ventral stream” areas (V2, V3, V4) preferred moderate-speeds in the central visual field, while motion area MT+ responded equally well to both speeds at all eccentricities. In all other areas and eccentricities BOLD responses were significant and equally strong for both types of moving stimuli. Support vector machine showed that the direction of the fast-speed motion could be successfully decoded from the BOLD response in all visual areas, suggesting that responses are mediated by motion mechanisms rather than being an unspecific preference for fast rate of flicker. The results show that the visual cortex responds to very fast motion, at speeds generated when we move our eyes rapidly, or when moving objects pass by closely.

## Introduction

**Figure 1:**
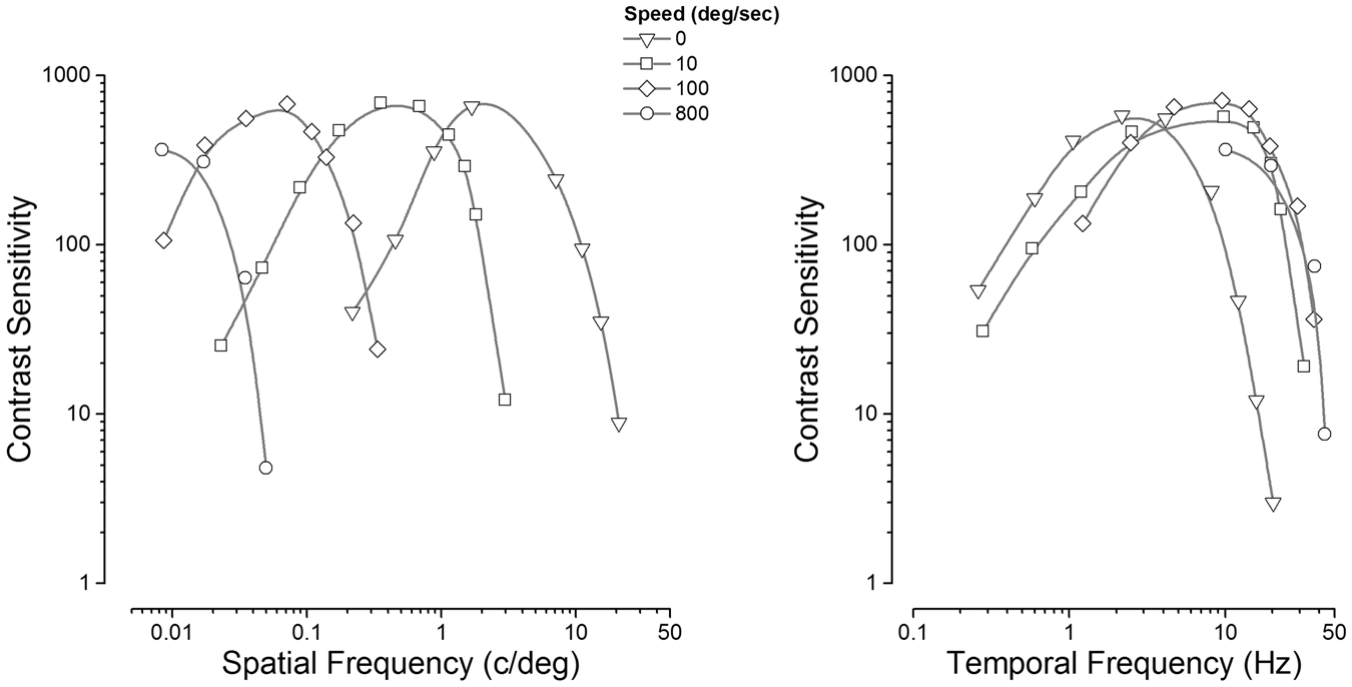
Human contrast sensitivity functions, measured with sinusoidal grating drifting at four different speeds. (**a)** Data plotted as a function of spatial frequency – the curves clearly shift towards lower frequencies at higher speeds. (**b)** Same data plotted as a function of temporal frequency – the curves are almost entirely superimposed. Adapted with permission from Burr & Ross^3^.

Motion perception has been studied extensively over the last half-century, with a variety of psychophysical, electrophysiological and neuroimaging techniques^1,2^. Most imaging and electrophysiological studies have used moderate speeds, usually below 10°/s, and very rarely above 100 °/s. However, humans and other animals can detect and discriminate far higher speeds with great sensitivity. For gratings of very low spatial frequency (<0.01 c/°), speeds of 10,000°/s can be reliably discriminated, whereas gratings of low spatial frequency (between 0.01 and 0.05 c/°) can be perceived at speeds of up to 1000 °/s, with no loss in sensitivity compared with lower speeds^3^. However, these low spatial frequencies are usually invisible when presented stationary or at low speeds (compare the contrast sensitivity functions of Fig. 1 represented with circles and with the triangles). Perceiving fast speeds is important, as saccadic eye-movements can create retinal motion at speeds of up to 800°/s^4,5^. Normally we are not aware of this motion during saccades^6–9^, implying that it is actively suppressed.

There has been a good deal of psychophysical work investigating the properties of neural mechanisms responding to fast motion. Burr and Ross^3^ showed that the limit of detecting motion is not velocity *per se*, but temporal frequency. Figure 1a shows measurements of contrast sensitivity for sinusoidal gratings as a function of spatial frequency for four different image speeds. The effect of image motion is not to decrease sensitivity but to shift the characteristic contrast sensitivity function down the spatial frequency scale: sensitivity to fast motion at low spatial frequencies is as high as for slow motion at high spatial frequencies. When the same results are plotted as a function of temporal frequency, the product of speed and spatial frequency, in Fig. 1b the curves tend to line up with each other, with none exceeding 50 Hz, pointing to a temporal-frequency limit of speed processing.

A series of studies using masking and summation techniques provided further evidence that motion is analyzed by a bank of detectors spanning a range of spatial frequencies but all with similar temporal frequency preference^10–13^. The preferred temporal frequency for motion detectors of all preferred speeds is around 10–12 Hz (depending on luminance), while the preferred spatial frequency varies over a much wider range, covering at least 0.06 to 30 c/deg^10^. There is also a great variability in the size of the summation fields of different preferred speeds, ranging from about 2 arcmin for low speeds, to 7 deg for high speeds.

While the psychophysics is quite clear, it is less clear what visual areas respond to such fast motion at low spatial frequencies. The best-known motion-selective area is V5/MT+, the human homolog of monkey areas MT and MST, which is responsive to a variety of moving stimuli such as random squares, gratings, dots, incoherent flicker and optic flow^14–16^. However, the cortical network mediating motion perception is quite extensive, comprising the primary visual cortex (V1), areas V2 and V3 and high associative and multisensory cortices such as the posterior vestibular insula^15,17–21^. The dorsal extrastriate area in humans, V3a, is highly motion-selective^22,23^, as is the adjacent V3b, sometimes referred to as the Kinetic Occipital region, which is selective to motion boundaries also generated by second-order motion^20,24–26^. Visual area V7 has also been shown to respond well to optic flow stimuli and even more strongly when a stereoscopic depth gradient was added to the stimulus^27^. Interestingly, many of the human areas have clear homologies in monkeys as assessed by their selectivity for coherent versus random dot kinematograms and to first- and second-order motion^28–31^. The role of subcortical structures such as the lateral geniculate nucleii and the superior colliculi in visual motion processing has received very little investigation to date.

As few large-field displays are MRI-compatible, most imaging studies concentrate on the central ± 15° of visual field^15,16,22,25^, and have used low to moderate speeds (<30 °/s). Compared with an eccentricity of ±1°, at ± 15° the receptive field sizes of primary visual cortex change only by a factor of three^32–34^, increasing the optimal speed by a similar amount. A few studies have used faster motion and far peripheral visual fields^35,36^, leading to the mapping of the human homolog of macaque visual areas V6 and V6A, which are sensitive to flow fields and translational ego-motion having a key role in the sensation of vection^35,37^. The precuneus and the cingulate sulcus have also been shown to process self-motion using wide-field stimulation^38–40^. However, none of these studies used stimuli of very low spatial frequency or very fast motion.

The aim of the current study is to use fMRI to investigate how rapid motion extending into the peripheral visual field (~ 60°) is processed. We contrasted the responses to two speeds of moving sinusoidal gratings: 38 and 570°/s, both at 10 Hz temporal frequency, which corresponds to optimal sensitivity^3^. The lateral geniculate nucleus, the superior colliculus and many cortical areas responded equally to botg;gh speeds. However, fast motion activated more strongly the far periphery of V1, but elicited no response in the central visual field of the ventral areas. To determine whether sinusoidal gratings moving at 570°/s are processed as real motion or unstructured flicker, we trained and tested a support vector machine classifier to discriminate the direction of motion. Classification accuracy in all visual areas under investigation was significantly above the 50% chance level, showing that indeed the response to sinusoidal gratings moving at such high speed is selective to the direction of motion and not simply to rate of flicker.

## Materials and Methods

### fMRI scanning

Scanning was performed with a GE 3T scanner (Excite HDx, GE Medical Systems, Milwaukee, WI) at the *Fondazione CNR/Regione Toscana G. Monasterio* in Pisa, Italy. The study was approved by the ethics committee of the *Azienda Ospedaliero-Universitaria Pisana* (protocol number 3255, approved on 20/01/2009) and was in accordance with the ethical standards of the 1964 Declaration of Helsinki. Ten healthy participants (3 female, 25–58 years old, all right-handed) with normal or corrected-to-normal visual acuity were scanned. Informed written consent was obtained from each participant prior to scanning sessions, in accordance with the guidelines of the MRI Laboratory. Each fMRI session consisted of six functional and one structural scans. Functional scans comprised three retinotopic (see Retinotopic Mapping section below) and three motion (two for cortical areas and one optimized for subcortical structures; for details see below). In four subjects we performed two additional scans with high-speed drifting gratings for the SVM analysis.

Three-dimensional (3-D) anatomical images were acquired at 1 × 1 × 1 mm resolution using a T1-weighted magnetization-prepared fast Spoiled Gradient Echo (SPGR) sequence (FOV = 256 mm, BW = 15.63, 256 × 256 matrix, TE = minimum full). For acquisition of retinotopic maps Echo Planar Imaging (EPI) sequence was used (FOV = 240 mm, 128 × 128 matrix, voxel size: 1.875 × 1.875 × 3 mm³, 19 axial slices, flip angle = 90°, TE = 30 ms, TR = 2500 ms). EPI sequence was also used for acquisition of cortical motion-sensitivity maps (FOV = 240 mm, 128 × 128 matrix, voxel size: 1.875 × 1.875 × 3 mm³, 19 axial slices, flip angle = 90°, TE = 30 ms, TR = 1500 ms). For seven out of ten participants we optimized the EPI sequence to obtain motion-sensitivity maps in sub-cortical structures, specifically the lateral geniculate nucleii and superior colliculi (FOV = 240 mm, 128 × 128 matrix, voxel size: 1.875 × 1.875 × 2 mm³, 19 axial slices, flip angle = 90°, TE = 30 ms, TR = 1500 ms). EPI sequence was also used for acquisition of responses to high-speed drifting gratings of opposite directions for SVM analysis for four out of ten participants (FOV = 240 mm, 128 × 128 matrix, slice thickness = 2.4 mm, 30 axial slices, flip angle = 90°, TE = 40 ms, TR = 3000 ms). The first 13 s of each functional acquisition were discarded from data analysis to achieve a steady state.

### Visual stimuli

Drifting gratings and conventional retinotopic mapping stimuli were generated on a VSG 2/5 Visual Stimulus Generator (Cambridge Research Systems) controlled by MATLAB programs (The MathWorks, Natick, MA) in conjunction with routines from the PsychToolbox^41,42^. All participants viewed stimuli monocularly through the right eye, with the left eye obscured by a black patch. Stimuli were projected through a custom-made magnetic-imaging-compatible visual projection system designed by our group to project on a very wide visual field^43^. Stimuli were projected in such a way so that the right visual field was stimulated up to ~60° and the left visual field up to ~25°. The resolution of the projection display was limited by the 10,000 of optic fibers, arranged within a circular window. The temporal resolution was 60 Hz.

### Drifting-gratings

We stimulated the visual cortex by presenting large-field (~ 60°) gratings drifting at moderate and high speeds. These stimuli have identical contrast (50%) and temporal frequency (10 Hz), differing only in speed and spatial frequency by a factor of 14. Spatial frequency for high-speed drifting gratings was 0.018 c/° at screen centre, corresponding to 571 °/s; for moderate-speed drifting gratings spatial frequency was 0.26 c/°, and speed 38 °/s. Given that the screen was flat, the spatial frequency (and hence speed) was not constant at the eye, but decreased with eccentricity. For stimuli in the far periphery (30–60°), spatial frequency and speed were about 40% lower on average than in the centre. However, this distortion was the same for both types of stimuli, and did not influence temporal frequency. Background luminance was 7 cd/m^2^. At this luminance the grating of 0.018 c/° was invisible when presented stationary. We used a block design, with the high-speed and the moderate-speed drifting gratings displayed alternately for a period of 15 s, alternating direction within each block every 2.5 s (starting leftward), each followed by a 15 s blank period. Each block was repeated six times. Static versions of the 0.018 c/° grating were invisible^3,44^ at 7 cd/m^2^, so we opted to contrast moving stimuli against blank periods, instead of static patterns as is often the practice for motion localizers.

We measured psychophysical contrast sensitivity for the two types of moving gratings using a single interval two-alternative forced-choice paradigm: on each trial (marked by a tone) the participant was asked to report whether a visual stimulus was moving leftwards or rightwards. Sixty trials were performed for each of four blocked stimulus conditions: fast-whole field, fast-periphery, moderate-whole field and moderate-periphery. Stimulus interval duration was 1 s; inter-trial interval was varying between 100 and 200 ms. Mean luminance was approximately 6 cd/m^2^. The psychophysical contrast sensitivity was determined on-line by the Quest algorithm^44^ homing in on the point where detection of the moving grating was 75%.

For the SVM classification analysis of motion direction, we presented three versions of the high-speed drifting grating: a leftward-moving, a rightward-moving and a stationary one. These were presented pseudo-randomly in a block design each lasting 12 s and each block was repeated ten times. The stationary grating stimulus was always below threshold, and the BOLD response were used as a baseline to calculate the response to motion.

### Attentional Task at central fixation

During the drifting-grating MRI scans, participants were asked to perform a sustained attentional task in central fixation. Specifically, the color of the fixation spot varied pseudo-randomly (on average once every four seconds) with small increments along the red, green and blue guns throughout the course of the experimental run; participants were asked to keep a mental count of the number of times the fixation dot was red. Mean accuracy for all participants was 98%.

### Retinotopic Mapping

Retinotopic maps were constructed using (i) horizontal and vertical meridian stimulation, (ii) upper, lower, left and right stimulation of the four visual quadrants and (iii) checkerboard ring stimuli to map eccentricity.

For (i) we used stimuli comprising 100 circular dots, half black and half white, moving on a grey background in two symmetrical sectors across the fixation point along the two principal meridians^45–47^. Each dot had a lifetime of 20 frames or 333 ms at a refresh rate of 60 Hz (local speed at linear trajectory = 6.5 degs^−1^). A block design was used with meridians stimulated interchangeably (6 repetitions) for 15 s and motion direction inverting seven times to avoid BOLD adaptation. For (ii) we used stimuli comprising 250 circular dots, half black and half white, moving on a grey background in four quadrants. A block design was used with quadrants stimulated sequentially clockwise, starting from upper right. All other information is identical to (i). To map the eccentricity of the visual cortex (iii), we presented three annuli comprising black and white checks at different eccentricities (contrast reversal at 4 Hz, contrast of 0.9) over a mean gray background. The outermost annulus had and an outer radius of 60° and an inner radius of 35° of visual angle. The medium-sized annulus had an outer radius of 27° and an inner radius of 20° and the central circular stimulus had 8° radius. Stimuli were presented sequentially for 15 s in a block design starting with the most eccentric stimulus, followed by the medium-sized and foveal stimulus (6 repetitions).

### Data analysis

Data were analyzed by Brain Voyager QX (Version 20.2, Brain Innovation, Maastricht, Netherlands) and MATLAB (MathWorks, MA). Prior to statistical analysis, functional data underwent pre-processing steps including 3-D motion correction, linear trend removal, and high pass filtering. Slice scan-time correction was performed for functional data.

Functional data were co-registered on the 3D anatomical T1-weighted images by using a gradient-based affine alignment with the standard Brain Voyager nine parameters (three for translation, three for rotation and three for FOV scale). For each individual participant, anatomical and functional data were transformed first into their own AC-PC space (rotating the cerebrum into the anterior commissure – posterior commissure plane) and then into Talairach space. To generate inflated surfaces for each hemisphere the white–grey boundary was traced, using an automatic segmentation algorithm, supplemented by manual correction by an expert operator to correct errors generated by the automatic routine. This segmentation was also used to automatically reconstruct the surface of the outer grey matter boundary, which was subsequently inflated and flattened.

### Subcortical Regions of Interest (ROIs)

BOLD activity in subcortical regions anatomically consistent with the position of the LGN and the SC (see results section) was evaluated on an average brain created from combining multiple volumes (N = 7) using Brain Voyager QX (Version 20.2, Brain Innovation, Maastricht, Netherlands). The seven co-registered functional datasets were used for a multi-subject analysis. Specifically, a fixed–effect (FFX) General Linear Model-based analysis at the statistical threshold of q(FDR) < 0.05 (no cluster thresholding) was performed after co-registration in Talairach space. For each subject, BOLD responses were analysed using a GLM, modeling the regressor of interest, by convolving a box-car function for each stimulation block with a gamma variate function for the hemodynamic response.

One-way repeated measures Analysis of Variance (ANOVA) was conducted to compare activity to fast- and moderate-speed gratings on each one of the subcortical ROIs (right LGN, left LGN, SC) defined as the combined response of both stimuli against blank. Effect size, r, is calculated as

1 $$\sqrt{1-(\frac{{\sum }_{({{\rm{y}}}_{{\rm{i}}}-{{\rm{\beta }}}_{0}-{{\rm{\beta }}}_{1}{{\rm{x}}}_{{\rm{i}}})}2}{{\sum }_{({{\rm{y}}}_{{\rm{i}}}-{{\rm{y}}}_{{\rm{mean}}})}2})}$$

where ${{\rm{y}}}_{{\rm{i}}}$ is BOLD modulation, ${{\rm{\beta }}}_{0}$ and ${{\rm{\beta }}}_{1}$ are the regression coefficients, and ${{\rm{x}}}_{{\rm{i}}}$ the time points.

### Evaluation of fMRI activity in cortical areas

Individual retinotopic maps were created to identify areas V1, V2, V3, V3a, V3b, lateral occipital complex (LOC), V4, MT+, V6 and V7. The procedure we followed comprised conventional retinotopic mapping procedures using the vertical and horizontal meridians followed by contrasting (a) Fovea vs. Periphery & Extreme Periphery, (b) Periphery vs. Fovea & Extreme Periphery, and (c) Extreme Periphery vs. Fovea & Periphery, to determine foveal, peripheral and far peripheral representations of each independent visual area. This allowed to subdivide each visual area according to its visual field eccentricity representation (0–15°, 15–30°, 30–60°). Figure S1. demonstrates how the eccentricity data were used to establish the position of the cyan and white border lines (see results section). To define V3a and V3b, we used the lower vertical meridian as the boundary between V3 and V3a and the upper vertical meridian as the boundary between V7 and both V3a and V3b. Within this area there were two distinct foveal representations, one more dorsal (V3a) and one more ventral (V3b), with increasingly peripheral representations running orthogonally to each other^48^. For each participant, BOLD responses were analysed using a General Linear Model (GLM) by convolving a box-car function for each stimulation block with a canonical hemodynamic response function (HRF). For each one of these ROIs, an ANOVA was conducted and the beta weights for the two types of motion (fast/moderate) were extracted for the left hemisphere only. We did not study the right hemisphere because the size of the projection screen allowed stimulation only up to 25° eccentricity.

### Support Vector Machine Analysis

We use in-built BrainVoyager QX v.20.2 routines to run a linear support vector machine (SVM) classifier with a fixed regularization parameter C = 1 for four participants. For each trial and voxel within the ROI of each visual area we estimated and z-normalized t-values using a two-gamma hemodynamic response function. These values were used for training and testing the linear SVM classifier. The classifier was trained with 70 trials, 35 for each direction of motion, and then tested with the 10 left-out trials (5 per direction). For each participant, this procedure was repeated 50 times, with different random draws of the 10 left-out trials. The average accuracy of the classifier across bootstraps was calculated for each participant and is reported as percentage correct.

To test the significance of the decoding accuracy, we ran a permutation bootstrap, where we shuffled the labels regarding the direction of motion (left- or right-drift) of the training trials, and tested decoding accuracy on the 10 left-out trials. We reiterated this procedure 1000 times and an estimate of significance was calculated (proportion of bootstrapped trails with accuracy greater than with original non-shuffled data).

## Results

### Psychophysics

**Figure 2:**
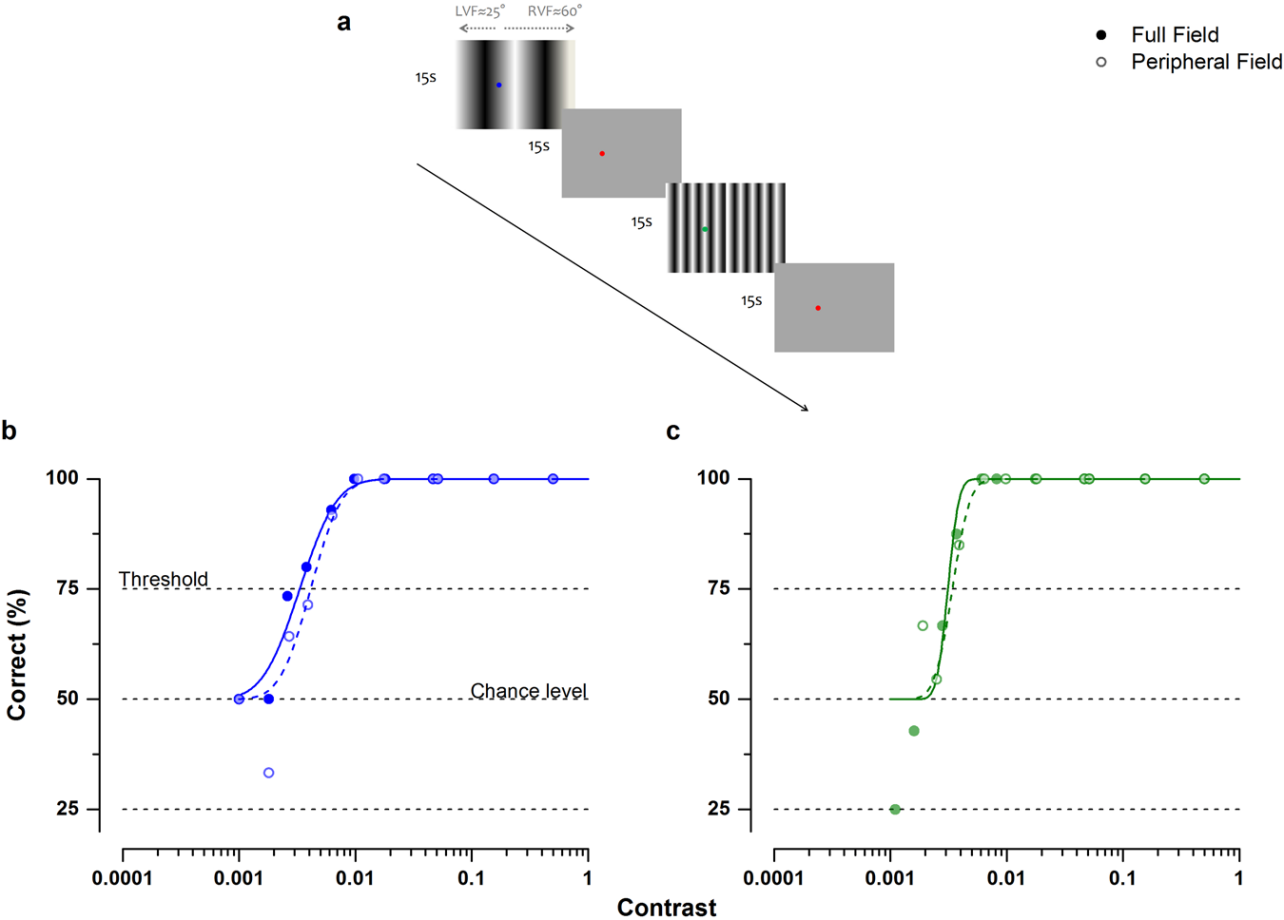
(**a**) Timeline of experiment illustrating the two types of moving-gratings used to stimulate the cortex. These stimuli have identical contrast envelopes (50%) and temporal frequency (10 Hz), differing only in speed and spatial frequency by a factor of 14. For more details see Materials & Methods section (**b** and **c**). Psychophysical contrast sensitivity functions for participant S8 for fast (**b**) and moderate-speed (**c**) stimuli, presented over 60 degrees of visual field (filled symbols), and confined to 30–60 degrees of visual field eccentricity (open symbols). Data were binned every 0.2 log-units. Solid lines show fits to filled dots (full field), whereas dashed lines show fits to open dots (peripheral field).

Figure 2 shows the psychometric functions for measuring contrast sensitivity for the fast (b) and moderate-speed gratings (c) presented on the full field and between 30 and 60 degrees of eccentricity for one example subject, who was asked to report the direction of a moving grating (left or right). The data points show percent-correct responses, which were fitted with a cumulative gaussian function: threshold was taken as 75% correct. Contrast sensitivity (inverse of threshold) was very similar for all types of gratings; for the high-speed gratings with full-field and peripheral stimulation it was ≈300 and ≈240 respectively, whereas for the moderate-speed gratings with full-field and peripheral stimulation psychophysical contrast sensitivity was it was ≈320 and ≈300 respectively. At 50% contrast (that used during fMRI sessions), detection of both types of gratings was 100%, both for the full-field and the peripheral presentation. Any differential response to the two gratings cannot be a trivial consequence of a difference in visibility or contrast, but implies a difference in the selectivity of the responding mechanisms.

**Figure 3:**
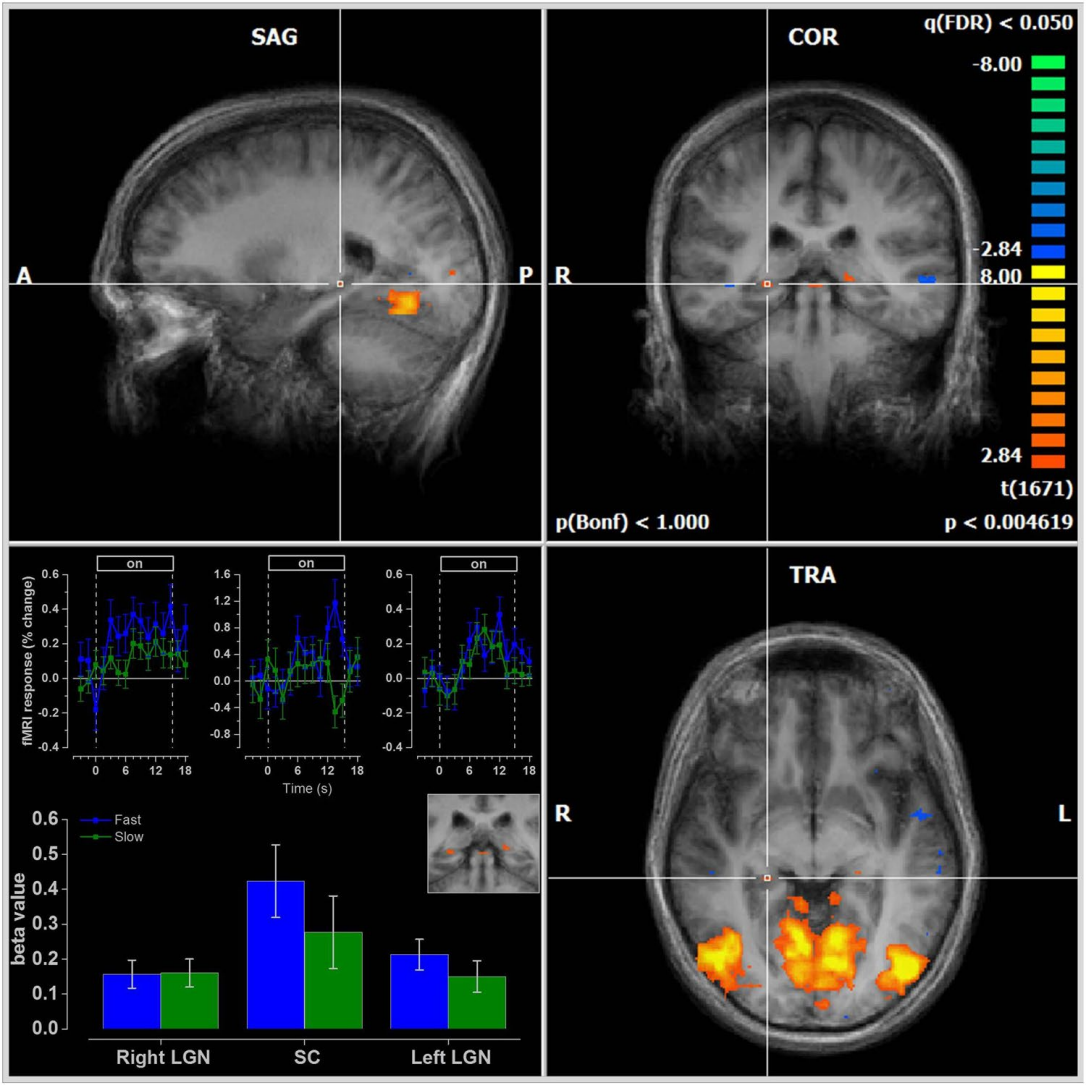
Anatomical regions consistent with the position of the LGN (TAL - LH: X = 21, Y = −27, Z = −4; RH: TAL: X = 21 Y = −28 Z = −2) and the SC (TAL: X = −2 Y = −31 Z = −5) projected on an average anatomy respond equally well to both speeds (N = 7). The cross hairs indicate the right LGN. The average time-series for the LGN and SC are shown in the lower left panel. The bars plot the estimated β-values from multi-subject GLM analysis. The SC are most clearly seen in the inset in the lower left panel, above the bar plot.

### Sub-cortical structures

Figure 3 shows responses to fast and moderate speed gratings overlaid on the average anatomy using a higher resolution EPI sequence, optimized for sub-cortical structures (*N* = 7). There is a clear and highly significant response to these stimuli compared with blank stimulation in an anatomical region consistent with the position of LGN and SC. The one-way repeated measures ANOVA for the LGN and SC showed that the BOLD modulation is strong (LGN-R: F(2, 1671) = 11.8, *p* < *0.001*, r = 0.12; LGN-L: F(2, 1671) = 13.3, *p* < *0.001*, r = 0.13; SCs: F(2, 1671) = 9.3, *p* < *0.001*, r = 0.11). The fMRI activity to fast (LGN-R: β = 0.157, t(1680) = 3.9, *p* < *0.001*; LGN-L: β = 0.213, t(1680) = 4.790, *p* < *0.001*; SCs: β = 0.423, t(1680) = 4.09, *p* < *0.001*) and moderate-speed gratings (LGN-R: β = 0.160, t(1680) = 3.993, *p* < *0.001*; LGN-L: β = 0.150, t(1680) = 3.377, *p* < *0.001*; SCs: β = 0.277, t(1680) = 2.673, *p* = *0.007*) did not differ in any of the ROIs (LGNR: *p* = *0.952;* LGNL: *p* = *0.224;* SCs: *p* = *0.224*). The equality of the subcortical response to the two stimuli is consistent with the equal visibility of the stimuli and suggests that the response of the M- and P- pathways (with the later not projecting to SC) to the two stimuli is balanced (see Discussion).

**Figure 4:**
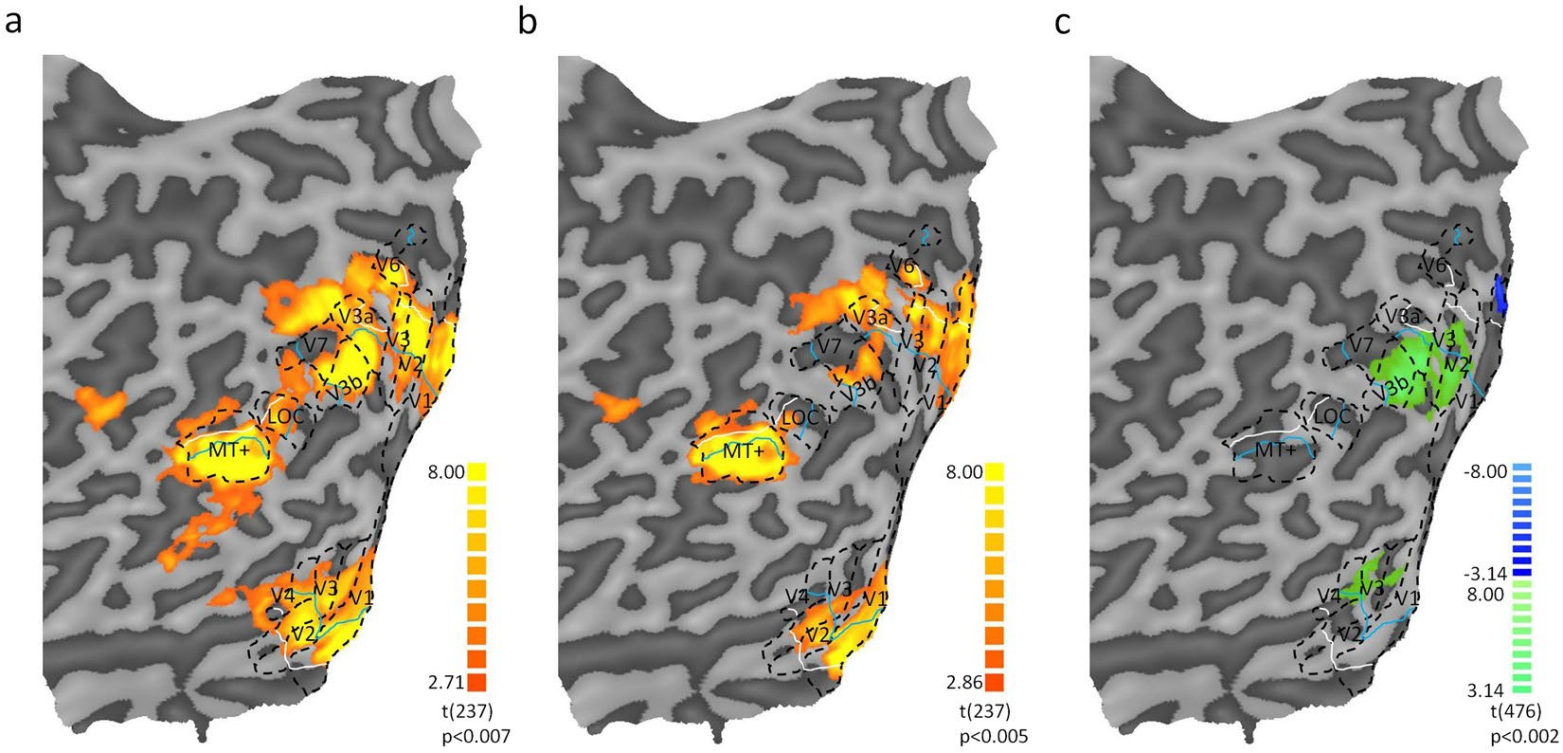
Left hemisphere flat maps showing t-statistics of a representative subject (S5). Dashed black lines represent boundaries of different visual areas; cyan lines indicate the boundary between central-peripheral visual field; white lines indicate the boundary between periphery-far periphery. (**a**) Typical pattern of cortical activation observed across visual areas to moderate-speed gratings and (**b**) fast-speed gratings (**c**). Contrast map showing preferential activation to fast (blue) or moderate-speed (green) gratings. Due to lack of coverage during functional scans, area V7 was not identified in participant S6.

### Cortical areas

For each participant we delineated the boundaries of visual areas V1, V2, V3, V3a, V3b, LOC, V4, MT+, V6 and V7 to create retinotopic maps (for details and example see Greco *et al*.^43^). Each visual area was subdivided into three sections using the eccentricity annuli: central (0–15°), periphery (15–30°) and extreme periphery (30–60°). Figure 4 shows left hemisphere flat mesh surfaces for participant S5 in response to (a) moderate- and (b) fast-speed gratings against blank, Fig. 4c shows contrast between moderate and fast-speed gratings. The response is significant both in central and in far periphery to both motions for most areas along the dorsal and ventral visual pathway, although it is stronger outside the central visual field, probably because of the low spatial frequencies of both stimuli. The BOLD GLM contrast shows that for most of cortex the activities are well balanced, with three exceptions: the central visual field representation of areas V2, V3, V3a, V3b and V4, the peripheral visual field representation of these same areas (except V3b) which show preferential activation to moderate-speed gratings (green color), and the far periphery of V1 which shows preferential activation to fast-speed gratings (blue color). We point out that this response was not produced by area prostriata, which has been described anatomically and functionally in humans^49,50^, and non-human primates^51^, where its properties are consistent with a preference for fast motion. Whereas both the extreme periphery of V1 and area prostriata show a preference for fast-speed gratings, the difference between the two is that the former shows significant, albeit reduced, activation to moderate-speed gratings too (t(9) = 4.87, *p* = *0.0009* vs. Figure 3B in Mikellidou *et al*.^49^).

**Figure 5:**
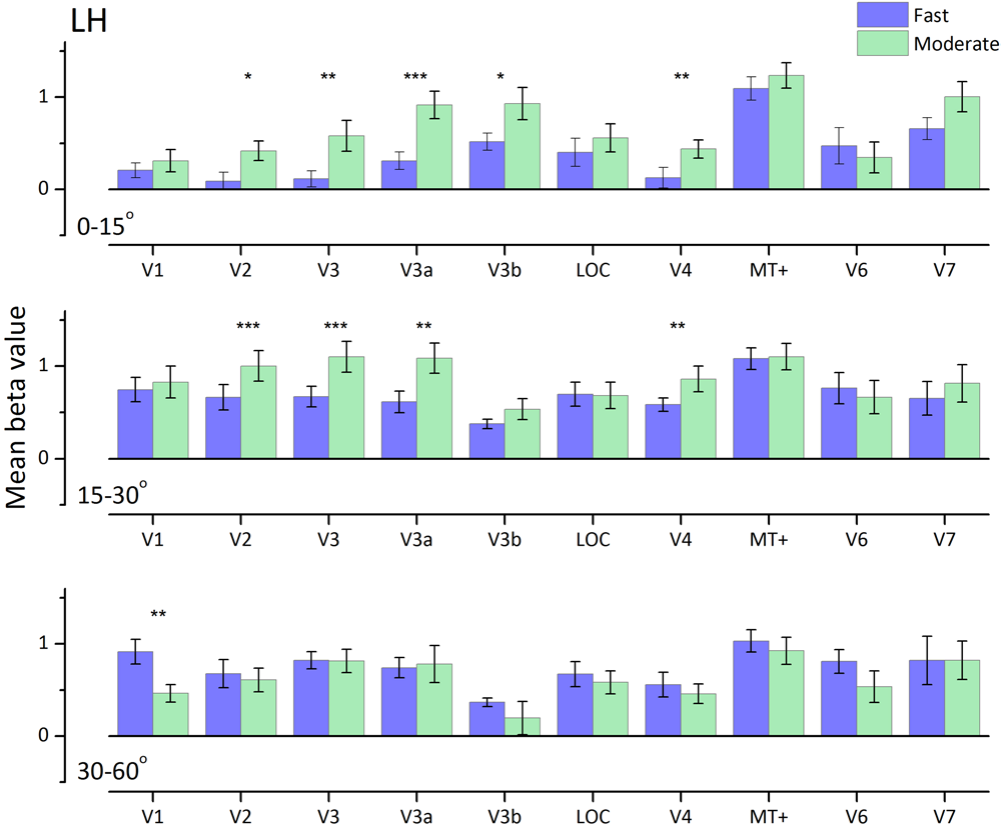
Average BOLD amplitude (mean beta values) and error bars (standard error of the mean) for the ROIs to fast- and moderate-speed gratings (blue and green respectively) at three eccentricities across participants for dorsal and ventral areas (*N* = 10). We used the Holm**’**s Sequential Bonferroni adjustment to correct for multiple comparisons. For 0–15°, *V2: p* = *0.014*; V3: *p* = *0.004*; V3a: *p* = *0.0003*; V3b: *p* = *0.012* V4: *p* = *0.008*. For 15–30°, V2: *p* = *0.0006*; V3: *p* = *0.0008*; V3a: *p* = *0.003*; V4: *p* = *0.008*. For 30–60°, V1: *p* = *0.002*.

Figure 5 illustrates average cortical responses from ten participants to both motion speeds at the three eccentricities. The beta values of all voxels in each ROI were calculated first for each individual, then averaged across individuals. We used the Holm’s Sequential Bonferroni adjustment to correct for multiple comparisons. While V1 responses from the central visual field representation to the two types of motion were significant and balanced, areas V2, V3, V3a, V3b and V4 showed a distinct preference for moderate-speeds (Fig. 5-first row; Table 1), with areas V2, V3 and V4 showing no significant response to fast-speed gratings (**V2**: t(9) = 0.942, *p* = 0.185; **V3**: t(9) = 1.27, *p* = *0.117*; **V4:** t(9) = 1.14, *p* = 0.142). At eccentricities of 15–30° (Fig. 5-second row; Table 1) areas V2, V3 and V4 respond to fast motion, but like V3a, they still prefer moderate-speed gratings. All areas except V1 show balanced responses to both types of stimuli at far peripheral eccentricities of 30–60°. Interestingly, V1 far periphery is the only area with statistically significant greater responses to fast motion (t(9) = 4.02, *p* = *0.002*). MT+, area V6 and V7 all showed strong and equal responses to both speeds of moving stimuli at all eccentricities.

**Table 1:** Mean beta values and standard deviation (*N* = 10) for cortical areas showing differential activation for fast compared to moderate-speed gratings.

Eccentricity	Cortical area	Mean beta (Standard Deviation)		
		Fast	Moderate	Paired T-test
0–15°	V2	0.090 (0.301)	0.417 (0.335)	t (9) = 2.61, *p* = *0.014*
	V3	0.114 (0.282)	0.580 (0.530)	t (9) = 3.40, *p* = *0.004*
	V3a	0.310 (0.305)	0.915 (0.469)	t (9) = 5.04, *p* = *0.003*
	V3b	0.516 (0.296)	0.930 (0.555)	t (9) = 2.72, *p* = *0.01*
	V4	0.126 (0.351)	0.438 (0.314)	t (9) = 2.94, *p* = *0.008*
15–30°	V2	0.664 (0.430)	1.00 (0.505)	t(9) = 4.69, *p* = *0.0006*
	V3	0.669 (0.353)	1.10 (0.531)	t (9) = 4.47, *p* = *0.0007*
	V3a	0.613 (0.374)	1.09 (0.517)	t (9) = 3.56, *p* = *0.003*
	V4	0.583 (0.228)	0.860 (0.441)	t (9) = 2.99, *p* = *0.008*
30–60°	V1	0.915 (0.424)	0.465 (0.301)	t (9) = −4.02, *p* = *0.002*

We used the Holm’s Sequential Bonferroni adjustment to correct for multiple comparisons.

**Figure 6:**
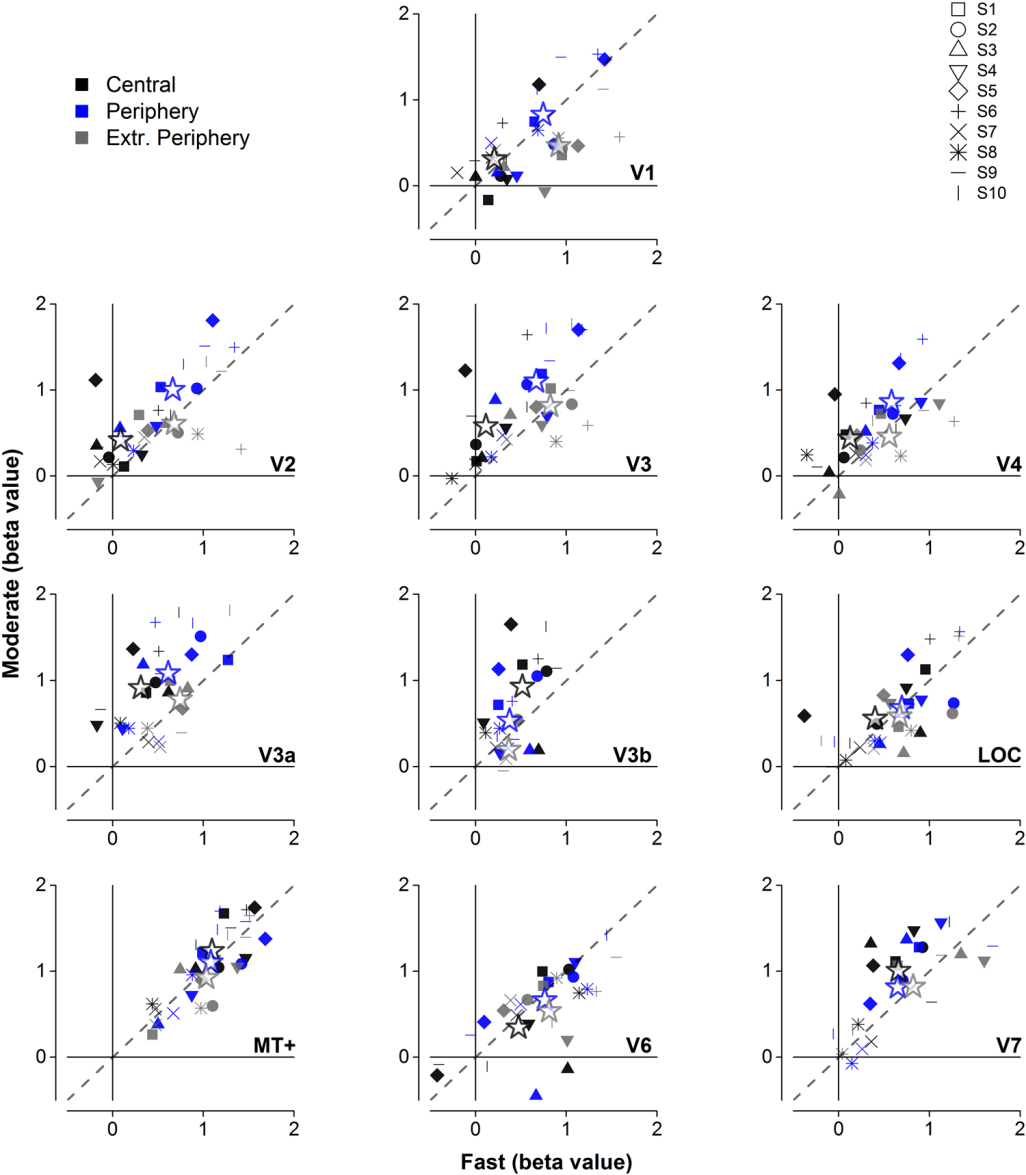
Scatterplots for all mapped visual areas showing individual beta values to fast- (abscissa) against moderate-speed (ordinate) gratings at 0–15° (black), 15–30° (blue) and 30–60° (grey). Symbols of the same shape represent a single participant. Dotted diagonal line shows the equality line. Each participant contributed three data points in each panel with the exception of missing values due to lack of coverage during functional scans (V3a Extreme Periphery: 3 participants, V3b Extreme Periphery: 7 participants, V6 Fovea: 1 participant, V6 Periphery: 1 participant, V7 Fovea: 1 participant, V7 Periphery: 1 participant, V7 Extreme Periphery: 4 participants). Areas of ‘ventral’ stream (V2, V3, V4) prefer moderate-speed gratings especially within 30° with most values lying over the equality line, whereas areas of the ‘dorsal’ stream (MT+, V6) are equally distributed over and above the equality line showing balanced responses to both types of stimuli.

To attempt to understand better the responsiveness of each area to high and moderate motion at the various eccentricities, and to report the data of individual subjects, we plot in Fig. 6 for each visual area and eccentricity the individual BOLD responses to moderate motion against those to fast. The various symbols refer to individual subject responses (color-coded for eccentricity), and large stars to values averaged over subjects. If BOLD responses were equally strong for fast and moderate motion at all eccentricities all points should fall near the equality line. To test this hypothesis, for each visual area we calculated the distance of each point away from the equality line using:

2 $$\frac{(fas{t}_{0}-moderat{e}_{0})}{\surd 2}$$

and then tested whether these are significantly different from zero by two-tailed one-sample t-tests. Zero would imply that a specific point lies on the equality line, a positive value a preference for fast motion and a negative value a preference for moderate motion. Areas of the ventral stream showed a significant preference for moderate-speed gratings (V2: t(29) = −2.72, *p* = *0.011;* V3: t(29) = −3.79, *p* = *0.0007;* V4: t(29) = −2.50, *p* = *0.018*), as well V3a (t(26) = −4.90, *p* = *0.00004*), V3b (t(22) = −2.45, *p* = *0.022*) and V7 (t(23) = −2.47, *p* = *0.021*). On the other hand, responses from the motion complex MT+ lie at all eccentricities almost perfectly along the equality line, showing no particular preference (t(29) = −0.37, *p* = *0.71*). Similarly, V1(t(29) = 1.20, *p* = *0.240*), V6(t(29) = 1.96, *p* = *0.060*) and LOC (t(29) = −0.27, *p* = *0.787*) show no significant preference overall.

**Figure 7:**
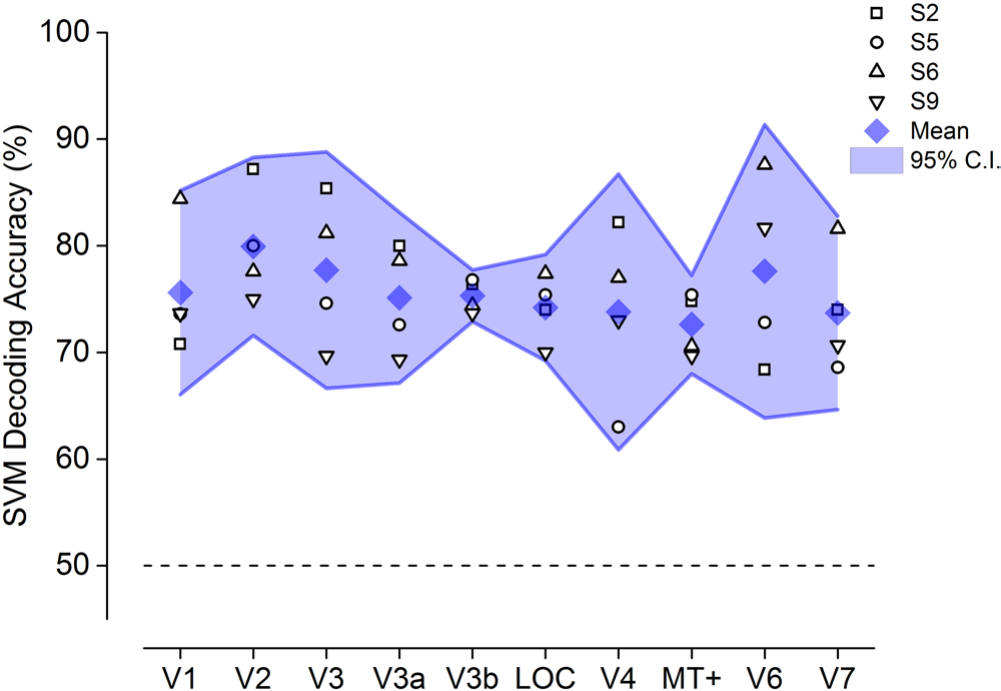
Support Vector Machine classification accuracies in decoding the direction of motion (left or right) of fast-speed gratings for all visual areas (*N* = *4*). The performance is significantly above chance (dashed line at 50%) for all areas. Shaded area shows 95% confidence intervals (C.I.).

The strong and unpredicted response to very fast motion, particularly for the ventral stream areas, could in principle originate from a response to fast flicker rate, rather than a real selectivity for motion. To test if these areas are selective to the direction of motion, we used a Multi-voxel pattern analysis (MVPA) technique to test whether the direction of fast-speed gratings can be successfully decoded from the voxel population of the visual areas under investigation. Figure 7 shows that Support Vector Machine (SVM) classification accuracy was significantly above chance for all four subjects in all visual areas, assessed by bootstrap permutation test (*p* < *0.05*).

### Microsaccadic Eye Movements

To rule out the possibility that our results reflect differences in eye movement direction, rather than differences in stimulus motion direction, we recorded microsaccadic eye movements outside the scanner in three participants (see.Fig. S2), using exactly the same drifting gratings, with direction reversal every 2.5 s, and sustained attention task in central fixation as in the initial fMRI experiment. On average, for each condition, microsaccades occurred for less than 1% of the time (Fast Left (FL): 0.61%; Fast Right (FR): 0.76%; Moderate Left (ML): 0.80%; Moderate Right (MR): 0.97%; Blank following Fast period (BLF): 0.50%; Blank following Moderate period (BLM): 0.51%), unlikely to be the basis of the direction decoding.

Nevertheless, we examined whether the horizontal direction of the microsaccades varied systematically with the direction of motion, at either speed. We made paired comparisons with two-tailed t-tests to compare independently the mean negative (leftward microsaccades) and positive (rightward microsaccades) tails of each motion condition with the corresponding blank period measured after that motion speed. No comparison revealed any significant differences across conditions, even without statistical corrections for multiple comparisons (FL− vs. BLF: t(7) = −1.99, *p* = *0.09*; FL+ vs. BLF: t(7) = −0.90, *p* = *0.40*; FR- vs. BLF: t(7) = −1.13, *p* = *0.30*; FR+ vs. BLF: t(7) = 1.16, *p* = *0.28*; ML− vs. BLM: t(7) = 1.19, *p* = *0.27*; ML+ vs. BLM: t(7) = 0.49, *p* = *0.64*; MR− vs. BLM: t(7) = 0.71, *p* = *0.50*; MR+ vs. BLM: t(7) = −0.65, *p* = *0.53*). Overall, the results show that the size, direction and number of microsaccades occurring for both types of drifting gratings, in either direction, are comparable with those during the mean luminance blank period, albeit some slight consistent left asymmetry across all conditions. Therefore, differential response to the two gratings is not a trivial consequence of microsaccadic eye movements, but implies a difference in motion selectivity.

## Discussion

Using fMRI and a custom-made optic fiber setup to stimulate the very far peripheral visual field, we investigated which visual areas respond to rapid motion at different eccentricities. All visual areas under study, both cortical and sub-cortical, responded well to both moderate and fast speeds.

Although all areas responded to the fast-moving, low spatial-frequency patterns, at least in the periphery, there was a clear difference in the pattern of responses between areas of the dorsal and ventral streams. Areas normally considered part of the dorsal stream – MT+, V6 and V7 – responded equally well to fast and moderate motion at all eccentricities. On the other hand, V2, V3, V3a, V3b, V4 and LOC – the last two considered part of the ventral stream – all preferred moderate motion within the central 15°. Indeed V2 and V4 showed no significant response to fast motion in central vision. For more peripheral stimulation, however, the responses were well-matched in all areas.

These results are consistent with the equal visibility of the two stimuli. While it was expected that the two stimuli would stimulate much of the brain, in principle a clearer dichotomy could have occurred between ventral and dorsal areas for the preference to the two stimuli. Previous psychophysical studies clearly demonstrate that velocity preference and tuning is a consequence of different size receptive field, with larger RFs preferring higher velocities and lower spatial frequencies, provided that the stimulus has the same temporal frequency^10,52^. In other words, all visual mechanisms tend to have the same temporal frequency selectivity but different preferred spatial frequencies, resulting in different velocity preferences. The present BOLD result reinforces the psychophysical evidence, and suggests that in all areas, with the few exceptions of central V2, V4 and LOC, there exist neurons that respond well to very large stimuli subtending up to 60 degrees of visual field.

The strong response to large, fast-moving stimuli is likely to reflect the existence of neurons with very large receptive fields^52^. Thus it is likely that these receptive fields are also present in the central visual field of V1, given the strong response to stimuli of 0.001 c/deg. The performance of the SVM classifier in all visual areas was significantly above the 50% chance level, demonstrating that direction of motion of fast-speed gratings can be successfully decoded. This result shows that BOLD responses were indeed driven by a response to visual motion and not simply by flicker. This is the first fMRI demonstration showing that the direction of such *ultra-fast* motion can be successfully decoded in the human visual cortex. Overall, the data clearly indicate that high speeds are analyzed throughout the entire visual brain.

The two subcortical areas, the superior colliculus and lateral geniculate body, responded equally well to both speeds of motion and spatial frequency. The fact that we observed equal responses in the LGN and SC, which receive quite different retinal input, suggests that the global response of each individual pathway elicited by these two motion stimuli is similar at all levels. The LGN receives input from the M, P and K streams, which project to segregated layers^53^. As far as it is known, the SC receives M and K input^54–56^. Most evidence from non-human primates suggests that the P-stream would be less sensitive to the fast than to moderate speeds at spatial frequencies lower than 1.5 c/° ^57,58^. In humans evidence is scant. However, a selective deficit for high-speed motion perception has been described in patients affected by an optic chiasm tumor and ocular Glaucoma, which affects mainly the M pathway^59,60^. It is therefore surprising that both LGN and SC gave balanced responses to the two image speeds, despite the over-representation of the P-cell population in the LGN. However, it is not clear how much each type of cell contributes to the BOLD response. The contrast response gains of the two populations are very different, with a higher gain for M-pathways^57^.

The activity in the LGN (Fig. 2) and V1 (Fig. 4 up to 30°) to both types of moving stimuli suggests that the global average responses to both speeds are balanced and may be propagated to higher order areas in similar way. Consequently, any preferential processing observed in higher-order areas is more likely to arise from the functional specificity and the intra-cortical processing of the area itself, or from the selective projection of a subpopulation of neurons. The selective projection interpretation would suggest that the lack of a significant response from the central visual field of ventral areas, which is the major target of the parvocellular pathway^61^, reflects the paucity of M inputs. It would also indicate that all areas receive in their peripheral visual field representation a strong input from M pathways and a decreased input from P pathways. This interpretation is consistent with the strong M projection observed in area V4 of the macaque monkey^62^.

The V3a is usually considered part of the dorsal pathway, but also showed a preference for moderate speeds. V3b is an area strongly implicated in the recognition of shape from motion, and is selective to second-order motion and kinetic contours^25,26^. Our data on motion speed preference would be consistent with the notion that both these areas use motion information for the recognition and categorization of object qualities, like other ventral areas^63,64^, and may have a stronger input from P-pathway.

The equality of responses observed in dorsal areas at all eccentricities may suggest that the response is mainly derived from M-inputs in these areas, obliterating the contribution from the P-input, which should show a central-peripheral gradient. However, this is not consistent with the V1 preference for fast motion in the far periphery, making more likely the suggestion that both M and P pathways project at all those areas, in agreement with the electrophysiological data from the monkey^28–31^.

The interpretation based on functional specificity of the area would suggest that some spatial inhibition of the central visual field responses to fast motion is active specifically for the V2 and V4 areas. The functional significance of this inhibition may be related to the neuronal selectivity of V4 for spatial feature processing, rarely associated with fast motion. However, this explanation would fail to explain the difference in response between central and peripheral visual field.

From the previous consideration it is clear that different factors contribute to the pattern of responses that we observed at various eccentricities. However, all the visual areas studied responded well to the very fast speed of 570 °/s, at least in the periphery. We make 2–3 saccades every second, and each saccade generates fast, wide-field motion, ranging from 700 to 800 °/s^5^. The results of this study suggest that a large part of the brain would respond to this motion, on every saccade, unless there were mechanisms in place to dampen the response. As these speeds stimulate a wide range of visual areas, it would make sense for the suppression to occur early in the processing sequencing. Indeed much evidence in human, both psychophysical^7^ and electrophysiological^65^ suggests that inhibition occurs in V1 or earlier, probably the thalamus. Such evidence is in accordance with a distinct suppression of LGN and V1 BOLD activity during saccadic eye movements over a constant illumination field, to eliminate any spurious retinal motion effects^66^. Interestingly, as stimuli were presented closer to the saccadic onset, the amplitude of V1 BOLD responses decreased^67^, following a similar dynamic to psychophysical results^7^. It would be very interesting to measure the BOLD response to the fast motion stimuli used here during saccadic eye movements.

Apart from during saccades, when does such fast motion occur naturally? Fast retinal motion is most likely to occur for close objects. One example would be moving our own arms rapidly in front of our eyes, continuously exciting our retina in our peripheral visual field with our own arm movement. A typical arm lifting movement has an amplitude of about 120 degrees at a distance of 25 cm from the eye, and takes about 500 ms, corresponding to a speed of about 240°/s. Although these movements do not attract much attention, the system is clearly capable of resolving them. Another instance when such fast motion can occur naturally may be running through dense foliage, close to our eyes. Otherwise, if the objects are not very close, they would have to be moving extremely quickly. For example, at 3 meters, an object would have to travel in the fronto-parallel plane at 25 m/sec (90 km/hr) to create an image speed of 500 °/s. It is not obvious that this would happen often in natural conditions, except maybe while driving a car in the highway. The fastest moving animal, the cheetah, can run at 100 km/hr in short bursts, but rarely 3 meters away in the fronto-parallel plane. Fast motion is therefore mainly self-generated motion and it is surprising that so much of the brain should respond to it, at least in the periphery.

## Electronic supplementary material

Supplementary Information

## References

[1] Burr D, Thompson P (2011). Motion psychophysics: 1985–2010. Vision Research.

[2] Nakayama K (1985). Biological image motion processing: a review. Vision Research.

[3] Burr DC, Ross J (1982). Contrast sensitivity at high velocities. Vision Research.

[4] Bahill AT, Clark MR, Stark L (1975). The main sequence, a tool for studying human eye movements. Mathematical Biosciences.

[5] Carpenter, R. H. S. *Movements of the Eyes*. (Pion, 1988).

[6] Burr DC, Holt J, Johnstone JR, Ross J (1982). Selective depression of motion sensitivity during saccades. The Journal of physiology.

[7] Burr DC, Morrone MC, Ross J (1994). Selective suppression of the magnocellular visual pathway during saccadic eye movements. Nature.

[8] Ross J, Burr D, Morrone C (1996). Suppression of the magnocellular pathway during saccades. Behavioural brain research.

[9] Diamond MR, Ross J, Morrone MC (2000). Extraretinal control of saccadic suppression. The Journal of Neuroscience.

[10] Anderson SJ, Burr DC (1985). Spatial and temporal selectivity of the human motion detection system. Vision Research.

[11] Anderson SJ, Burr DC, Morrone MC (1991). Two-dimensional spatial and spatial-frequency selectivity of motion-sensitive mechanisms in human vision. Journal of the Optical Society of America A.

[12] Burr DC, Ross J, Morrone MC (1986). Seeing objects in motion. Proceedings of the Royal Society B: Biological Sciences.

[13] Hess RF, Snowden RJ (1992). Temporal properties of human visual filters: Number, shapes and spatial covariation. Vision Research.

[14] Tootell RBH, Reppas JB, Kwong KK (1995). Functional analysis of human MT and related visual cortical areas using magnetic resonance imaging. Journal of Neuroscience.

[15] Zeki S (1991). A direct demonstration of functional specialization in human visual cortex. The Journal of neuroscience: the official journal of the Society for Neuroscience.

[16] Morrone MC (2000). A cortical area that responds specifically to optic flow, revealed by fMRI. Nature neuroscience.

[17] McKeefry DJ, Watson JDG, Frackowiak RSJ, Fong K, Zeki S (1997). The activity in human areas V1/V2, V3, and V5 during the perception of coherent and incoherent motion. NeuroImage.

[18] Sunaert S, Hecke VP, Marchal G (1999). Motion-responsive regions of the human brain. Experimental Brain Research.

[19] Kamitani Y, Tong F (2006). Decoding seen and attended motion directions from activity in the human visual cortex. Current Biology.

[20] Smith AT, Greenlee MW, Singh KD, Kraemer FM, Hennig J (1998). The processing of first- and second-order motion in human visual cortex assessed by functional magnetic resonance imaging (fMRI). The Journal of neuroscience: the official journal of the Society for Neuroscience.

[21] Seiffert AE, Somers DC, Dale AM, Tootell RBH (2003). Functional MRI studies of human visual motion perception: texture, luminance, attention and after-effects. Cerebral Cortex.

[22] Tootell RBH, Mendola JD, Hadjikhani NK (1997). Functional analysis of V3A and related areas in human visual cortex. The Journal of Neuroscience.

[23] Singh KD, Smith AT, Greenlee MW (2000). Spatiotemporal frequency and direction sensitivities of human visual areas measured using fMRI. NeuroImage.

[24] Zeki S, Perry RJ, Bartels A (2003). The processing of kinetic contours in the brain. Cerebral Cortex.

[25] Oostende VS, Sunaert S, Hecke VP, Marchal G, Orban GA (1997). The kinetic occipital (KO) region in man: an fMRI study. Cerebral Cortex.

[26] Dupont P (1997). The kinetic occipital region in human visual cortex. Cerebral cortex (New York, N.Y.: 1991).

[27] Cardin V, Smith AT (2011). Sensitivity of human visual cortical area V6 to stereoscopic depth gradients associated with self-motion. Journal of Neurophysiology.

[28] Galletti C, Battaglini PP, Aicardi G (1988). ‘Real-motion’cells in visual area V2 of behaving macaque monkeys. Experimental Brain Research.

[29] Gegenfurtner KR, Kiper DC, Fenstemaker SB (1996). Processing of color, form, and motion in macaque area V2. Visual Neuroscience.

[30] Albright TD (1992). Form-cue invariant motion processing in primate visual cortex. Science.

[31] Shipp S, Zeki S (1984). Segregation of pathways leading from area V2 to areas V4 and V5 of macaque monkey visual cortex. Nature.

[32] Dow BM, Snyder AZ, Vautin RG, Bauer R (1981). Magnification factor and receptive field size in foveal striate cortex of the monkey. Experimental Brain Research.

[33] Desimone R, Ungerleider LG (1986). Multiple visual areas in the caudal superior temporal sulcus of the macaque. The Journal of comparative neurology.

[34] Felleman DJ, Essen DC (1987). Receptive field properties of neurons in area V3 of macaque monkey extrastriate cortex. Journal of Neurophysiology.

[35] Pitzalis S (2006). Wide-Field Retinotopy Defines Human Cortical Visual Area V6. Journal of Neuroscience.

[36] von Pföstl V (2009). Motion sensitivity of human V6: a magnetoencephalography study. NeuroImage.

[37] Pitzalis S, Fattori P, Galletti C (2012). The functional role of the medial motion area V6. Frontiers in behavioral neuroscience.

[38] Wada A, Sakano Y, Ando H (2016). Differential Responses to a Visual Self-Motion Signal in Human Medial Cortical Regions Revealed by Wide-View Stimulation. Frontiers in psychology.

[39] Cardin V, Smith AT (2010). Sensitivity of human visual and vestibular cortical regions to egomotion-compatible visual stimulation. Cerebral cortex (New York, N.Y.: 1991).

[40] Wall MB, Smith AT (2008). The representation of egomotion in the human brain. Current Biology.

[41] Brainard DH (1997). The psychophysics toolbox. Spatial vision.

[42] Kleiner M (2007). What’s new in Psychtoolbox-3. Perception.

[43] Greco V (2016). A low-cost and versatile system for projecting wide-field visual stimuli within fMRI scanners. Behavior Research Methods.

[44] Watson AB, Pelli DG (1983). QUEST: a Bayesian adaptive psychometric method. Perception & Psychophysics.

[45] Engel SA, Glover GH, Wandell BA (1997). Retinotopic organization in human visual cortex and the spatial precision of functional MRI. Cerebral Cortex.

[46] Sereno M (1995). Borders of multiple visual areas in humans revealed by functional magnetic resonance imaging. Science (New York, N.Y.).

[47] Wandell B, Brewer A, Dougherty RF (2005). Visual field map clusters in human cortex. Philosophical Transactions of the Royal Society B: Biological Sciences.

[48] Barton B, Brewer AA (2017). Visual Field Map Clusters in High-Order Visual Processing: Organization of V3A/V3B and a New Cloverleaf Cluster in the Posterior Superior Temporal Sulcus. Frontiers in Integrative Neuroscience.

[49] Mikellidou K (2017). Area Prostriata in the human brain. Current Biology.

[50] Glasser MF (2016). A multi-modal parcellation of human cerebral cortex. Nature.

[51] Yu HH, Chaplin TA, Davies AJ, Verma R, Rosa MGP (2012). A Specialized Area in Limbic Cortex for Fast Analysis of Peripheral Vision. Current Biology.

[52] Anderson SJ, Burr DC (1987). Receptive field size of human motion detection units. Vision Research.

[53] Wurtz, R. H. & Kandel, E. R. In Principles of neural science (eds E. Kandell, J. Schwartz, & T. Messel) Ch. 523–547, (McGraw-Hill, 2000).

[54] Schneider KA, Kastner S (2005). Visual Responses of the Human Superior Colliculus: A High-Resolution Functional Magnetic Resonance Imaging Study. Journal of Neurophysiology.

[55] Hoffmann KP (1973). Conduction velocity in pathways from retina to superior colliculus in the cat: a correlation with receptive-field properties. Journal of Neurophysiology.

[56] Isayama, T., Berson, D. M. & Pu, M. Theta ganglion cell type of cat retina. *Journal of Comparative Neurology* **417**, 32–48, 10.1002/(sici)1096-9861(20000131)417:1 32::aid-cne3 3.0.co;2-s (2000). 10.1002/(sici)1096-9861(20000131)417:1<32::aid-cne3>3.0.co;2-s 10660886

[57] Kaplan E, Shapley RM (1986). The primate retina contains two types of ganglion cells, with high and low contrast sensitivity. Proceedings of the National Academy of Sciences.

[58] Derrington AM, Lennie P (1984). Spatial and temporal contrast sensitivities of neurones in lateral geniculate nucleus of macaque. The Journal of physiology.

[59] Tassinari G, Marzi CA, Lee BB, Lollo DV, Campara D (1999). A possible selective impairment of magnocellular function in compression of the anterior visual pathways. Experimental Brain Research.

[60] Shabana N, Pérès VC, Carkeet A, Chew PTK (2003). Motion perception in glaucoma patients: a review. Survey of ophthalmology.

[61] Livingstone MS, Hubel DH (1988). Do the relative mapping densities of the magno-and parvocellular systems vary with eccentricity?. The Journal of Neuroscience.

[62] Ferrera VP, Nealey TA, Maunsell JH (1994). Responses in macaque visual area V4 following inactivation of the parvocellular and magnocellular LGN pathways. The Journal of Neuroscience.

[63] Tanaka K (1996). Inferotemporal cortex and object vision. Annual review of neuroscience.

[64] Ungerleider LG, Haxby JV (1994). ‘What’ and ‘where’ in the human brain. Current opinion in neurobiology.

[65] Thilo KV, Santoro L, Walsh V, Blakemore C (2004). The site of saccadic suppression. Nature neuroscience.

[66] Sylvester R, Haynes JD, Rees G (2005). Saccades differentially modulate human LGN and V1 responses in the presence and absence of visual stimulation. Current Biology.

[67] Vallines I, Greenlee MW (2006). Saccadic suppression of retinotopically localized blood oxygen level-dependent responses in human primary visual area V1. The Journal of Neuroscience.

