# Supplementary material for: Cortical BOLD responses to moderate- and high-speed motion in the human visual cortex
Source: Sci Rep. 2018 May 29;8:8357. doi: 10.1038/s41598-018-26507-0 (PMC5974286; doi:10.1038/s41598-018-26507-0)
Supplement: Supplementary file 1 — Supplementary Information [file 41598_2018_26507_MOESM1_ESM.pdf]

# Cortical BOLD responses to moderate- and high-speed motion in the human visual cortex

Kyriaki Mikellidou*[1], Francesca Frijia [2], Domenico Montanaro[2], Vincenzo Greco[3], David C. Burr [4,5] & Maria Concetta Morrone [1,6]

*[1]Department of Translational Research on New Technologies in Medicine and Surgery, University of Pisa, Italy; [2]Unit of Neuroradiology, Fondazione CNR/Regione Toscana G. Monasterio, Pisa, Italy; [3]Istituto Nazionale di Ottica, CNR, Florence, Italy; [4]Department of Neuroscience, Psychology, Pharmacology and Child Health, University of Florence, Italy; [5]Neuroscience Institute, CNR, Pisa, Italy; [6]Stella Maris Scientific Institute, Pisa, Italy*

**Supplementary Methods**

**Measurement of Microsaccades:**

To evaluate the effect of the stimuli on eye-movements, we used an EyeLink 1000 system (SR Research, Canada) to monitor two-dimensional eye position in a simulation session outside the scanner, with head restrained by chin-rest. Eye position was measured with an infrared camera mounted below a monitor screen (CRS Display ++ LCD Monitor, 70 × 40 cm), at a distance of 57 cm. Eye-tracking data from the right eye were acquired at 1000 Hz and streamed from the EyeLink to a PC through the EyeLink toolbox for Matlab[1]. The experimental room was illuminated only by the monitor screen. Gaze position values were output online by the EyeLink system (computed with internal algorithms) and we used Matlab to receive and store them. We also used Matlab's built-in routines for defining and analyzing microsaccadic eye movements.

Recording sessions lasted 360 seconds (similar to the fMRI sessions), during which the high-speed and the moderate-speed drifting gratings were displayed alternately for a period of 15 s, alternating direction within each block every 2.5 s (starting leftward), each followed by a 15s blank period. Each motion block was repeated six times. Two participants from the fMRI experiment (S1, S7) and a new additional participant (X1) were instructed to stare straight-ahead, trying to avoid eye, head, and body movements and to keep blinking to a minimum while performing the sustained attention task at central fixation. Prior to analysing the data, we identified and removed blinks and then concatenated blocks of 2.5s periods for each one of the four types of motion stimulation (i.e. fast leftwards, fast rightwards, moderate leftwards, moderate rightwards). For the two types of blank periods (blank following fast, blank following moderate), we concatenated blocks of 15s. Microsaccades were defined as saccades with a magnitude $<2^\circ$ [2]; anything less than 0.05° was considered an artifact and excluded. Velocity threshold detection was set to 15°/s.

**Supplementary Figures**

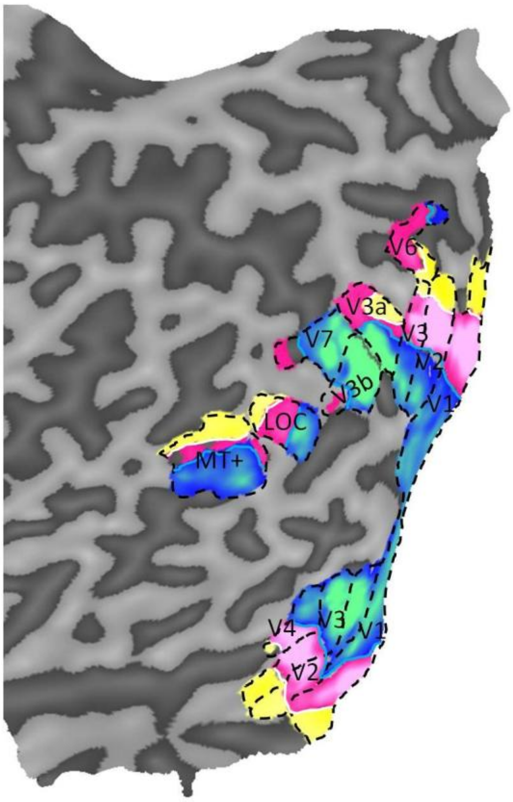


**Figure S1.**

Following conventional retinotopic mapping procedures using the vertical and horizontal meridians to define the borders between visual areas, we used eccentricity data to establish the position of the cyan and white border lines in Fig. 4 (see Methods for more details). This allowed to subdivide each visual area according to its visual field eccentricity representation (0-15˚, 15-30˚, 30-60˚).

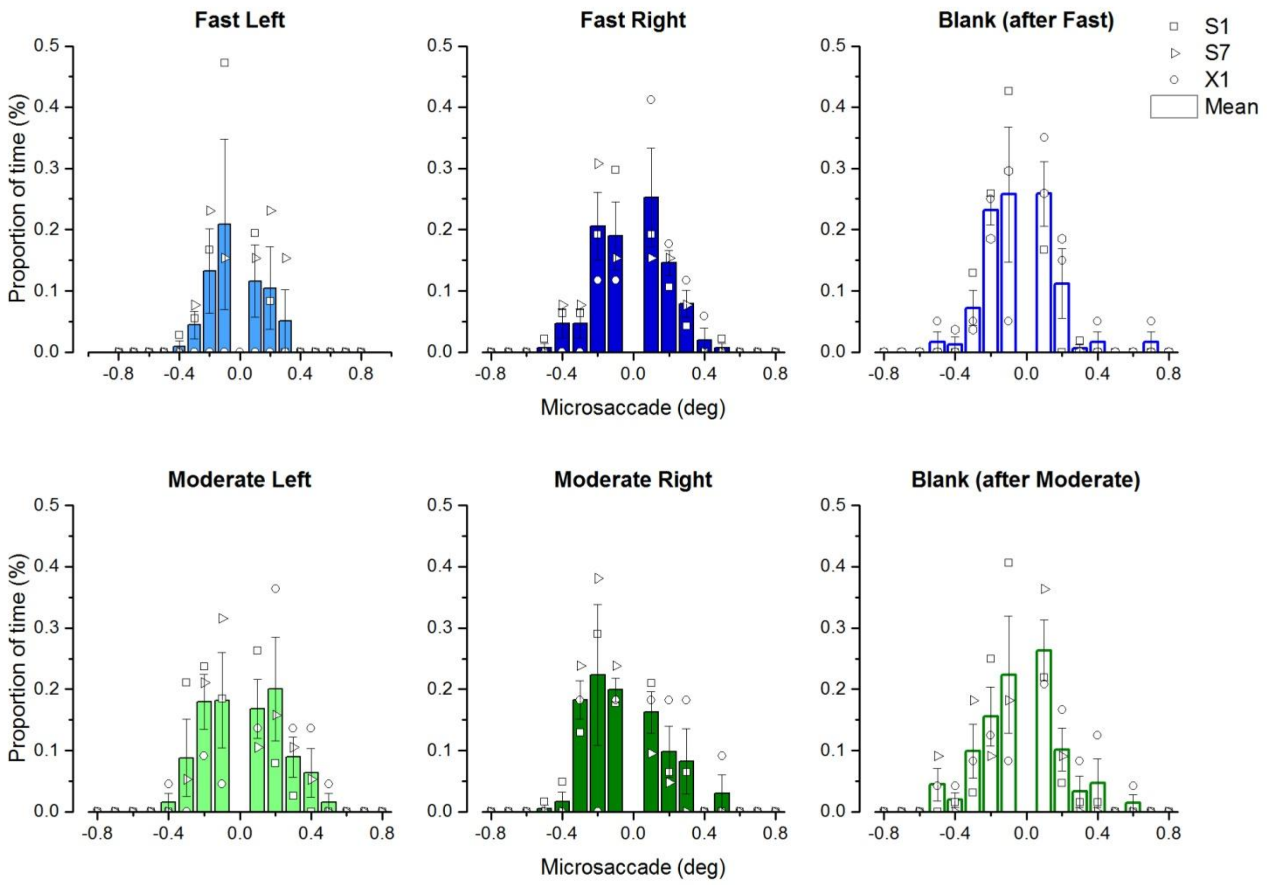


**Figure S2.**

Supplementary Figure 2. Recorded microsaccadic eye movement patterns for three participants (bars-mean; error bars- standard error of the mean; symbols-individual data) while participants were viewing high-speed and moderate-speed drifting gratings (see Methods for more details). Positive values indicate movement to the right and negative values indicate movement to the left. No consistent microsaccadic pattern was observed as a function of stimulus speed or direction.

## References

1 Cornelissen, F. W., Peters, E. M. & Palmer, J. The Eyelink Toolbox: Eye tracking with MATLAB and the Psychophysics Toolbox. *Behavior Research Methods, Instruments, & Computers* **34**, 613-617, doi:10.3758/bf03195489 (2002).

2 McCamy, M. B., Jazi, A., Otero-Millan, J., Macknik, S. L. & Martinez-Conde, S. The effects of fixation target size and luminance on microsaccades and square-wave jerks. *PeerJ* **1**, e9, doi:10.7717/peerj.9 (2013).
